# PID-controller enhanced artificial *β*-cells

**DOI:** 10.1371/journal.pone.0342799

**Published:** 2026-03-18

**Authors:** Lin Liu, Bruna Jacobson, Darko Stefanovic

**Affiliations:** 1 Department of Computer Science, University of New Mexico, Albuquerque, New Mexico, United States of America; 2 Department of Chemical and Biological Engineering, University of New Mexico, Albuquerque, New Mexico, United States of America; Université Paris Descartes, FRANCE

## Abstract

Conventional management of diabetes via injection or external insulin pumps suffers from inconvenience and inability to accurately maintain blood glucose levels. A potential solution to these problems consists of implanting synthetic artificial *β*-cells that can sense glucose and transcribe insulin protein. Experimental results from Xie et al. show these cells are able to release insulin and somewhat improve postprandial glucose levels in diabetic mice. However, they fail to achieve the degree of glucose regulation as in healthy mice. In our analysis, we explain that this artificial *β*-cell system has a major disadvantage: it is a high-dimensional dynamic system but with little tuning space. Here, we propose an analytical model of a PID-controller-based enhanced artificial *β*-cell design to solve this issue. Our numerical simulations show that a model of PID-controlled engineered artificial *β*-cells can shut down production of insulin in time and maintain a proper glycemia level, in addition to adding more tuning space. These enhanced models of PID-controller-based artificial *β*-cell are thus able to perform better in regulating glucose levels in Type 1 diabetic mice compared with artificial *β*-cells without PID-control.

## Introduction

Diabetes is a chronic illness that affects the body’s ability to convert food into energy [1]. Many mammalian species are susceptible to diabetes, including humans, mice, pigs, and dogs [2]. In a healthy body, carbohydrates are converted into glucose, which provides energy for daily activities. In response to an increase in blood glucose, *β*-cells in the pancreas release insulin. Insulin in turn allows blood glucose to enter cells so that it can be used as fuel. In diabetic animals, insulin production is insufficient or insulin is not utilized as efficiently as in healthy animals because the cells in muscles, fat, and liver do not respond to it. In either case, high blood glucose levels occur, which can lead to serious health problems, including heart disease, kidney disease, stroke, and lower limb amputations [3]. There are several phenotypes of diabetes, and the two most common are Type 1 diabetes (T1D) and Type 2 diabetes (T2D). While T2D involves insulin resistance, often associated with aging or obesity, T1D is an autoimmune disease marked by loss of functional *β*-cells leading to absolute or near absolute insulin deficiency. There is currently no prevention for T1D [4]. Patients with T1D must take in external insulin every day to maintain their blood glucose level in the normal range. Traditional treatment for diabetes includes oral medication intake, insulin injections, and insulin pumps, all of which suffer from inconvenience, inaccuracy, and hygiene issues such as risk of skin infection [5]. In 2016, Xie et al. proposed a novel approach for diabetic treatment, the artificial *β*-cell [6]. In particular, they introduce HEK-*β* cells that are genetically modified from the monoclonal HEK-293 cell line. Here we refer to these cells as artificial *β*-cells for short. According to this design, when there is a high concentration of glucose in the cell exterior environment, ATP concentration in the cell increases and subsequently ion channels are triggered. The final result of the ion channel activity is the transcription of mRNA and the subsequent production and secretion of insulin to lower blood glucose levels. Therefore, we can view the ion influx as a biological sensor of the glucose, and ATP as a real time indicator of glucose levels. Xie et al. also offer a detailed analytical model for these cells in a mouse model (equations and Fig S3 within S1 File). Artificial *β*-cells provide a degree of insulin regulation when implanted in a diabetic mouse; however, the regulation is not comparable to that in a healthy mouse. Our analysis of the model by Xie et al. shows that a plausible explanation lies in the low number of artificial *β*-cells implanted. However, if the number of artificial *β*-cells is increased, we find that a very high blood insulin level persists that is lethal to mice. A detailed analysis of the model indicated that this is due to an insufficiently sensitive signaling pathway from ATP to mRNA: the insulin production does not cease on time, even when the external glucose levels and the internal ATP levels are dangerously low. To mitigate this problem, here we present an updated model of an artificial *β*-cell that is based on the model by Xie et al. but which inserts an explicit controller into the signaling pathway, critically addressing the issues above.

Recent approaches to glucose regulation have largely focused on external device-based systems, such as the artificial pancreas, which combines continuous glucose monitoring with an external insulin pump controlled by algorithms including proportional-integral-derivative (PID) control, model predictive control (MPC), and fuzzy logic control to achieve closed-loop regulation in diabetic patients [7,8]. These external systems have demonstrated significant clinical success, but require implanted sensors, frequent recalibrations, and external hardware, limiting patient convenience and biological integration. In contrast, recent efforts in synthetic biology have explored embedding feedback control directly within engineered cells. Examples include gene circuits implementing integral feedback to achieve robust perfect adaptation in living cells [9], and designs of biomolecular PID controllers using chemical reaction networks [10]. While these studies have demonstrated the feasibility of implementing basic feedback mechanisms in biological systems, most existing biological controllers either focus on simple set point regulation without explicit derivative control, or have been validated only in abstract biochemical settings. Our work bridges these fields by proposing a full PID feedback control scheme embedded at the cellular level specifically for regulating glucose homeostasis, offering a new direction for biologically integrated diabetes therapy.

Having examined these limitations in the achieved performance of artificial *β*-cells, we explore via simulation what might be achievable if the section of the signaling pathway from ATP to mRNA were replaced by an explicit control circuit. Here we do not fully specify the design of that circuit, leaving it to a future paper, but we note that any such design will have to augment or modulate the machinery of the ion channels present in the cell. In choosing which control algorithm to realize first, a natural choice is simple feedback control, namely proportional-integral-derivative (PID) control, which has been used in external insulin pump control [11–13]. Therefore, we introduce a model for improving glucose level regulation using PID control in artificial *β*-cells. In principle, more advanced control algorithms, such as the Linear Quadratic Regulator (LQR), Model Predictive Control (MPC), and stochastic methods like the Kalman Filter, could be considered as well. However, their core operations—matrix multiplication and inversion—are not easily realized using chemical reaction networks inside a cell. Therefore we defer their consideration to future work.

PID control is also attractive because it has been shown that a chemical reaction network (CRN) with deterministic mass action semantics can implement integral, proportional, and derivative operations over chemical species concentrations, thereby implementing PID controller logic [10,14,15], wherein the integral, proportional, and derivative gain can be adjusted by appropriately setting the reaction rates. It is well established that chemical reaction rates within cells can be tuned and controlled [16–19]. In chemical reaction networks (CRNs), the dual-rail representation is a technique used to encode both positive and negative values by representing each quantity as a pair of molecular species. The value is interpreted as the difference between the concentrations of the two species, allowing the system to compute both positive and negative corrective actions—an essential feature for implementing PID control [10]. Here, we do not specify the components of such a CRN; instead we treat the PID controller abstractly and we focus on the feasibility of glucose level regulation with this class of controller, and the tuning of the PID gain parameters.

We show that the PID *β*-cells we propose could provide a more effective insulin production pathway. In the Methods and Results section, we show via computer simulation that PID *β*-cells can outperform the original model of artificial *β*-cells. Our model of T1D mice implanted with PID *β*-cells shows postprandial glucose level time courses closer to those of healthy mice, compared with T1D mice implanted with the original artificial *β*-cells without PID control. Moreover, the PID *β*-cells can also provide more tuning space on the glucose time courses compared with artificial *β*-cells, by varying controller parameters. In the Discussion section we touch on the advantages and the limitations of PID *β*-cells.

### Background on artificial *β*-cells

**Fig 1:**
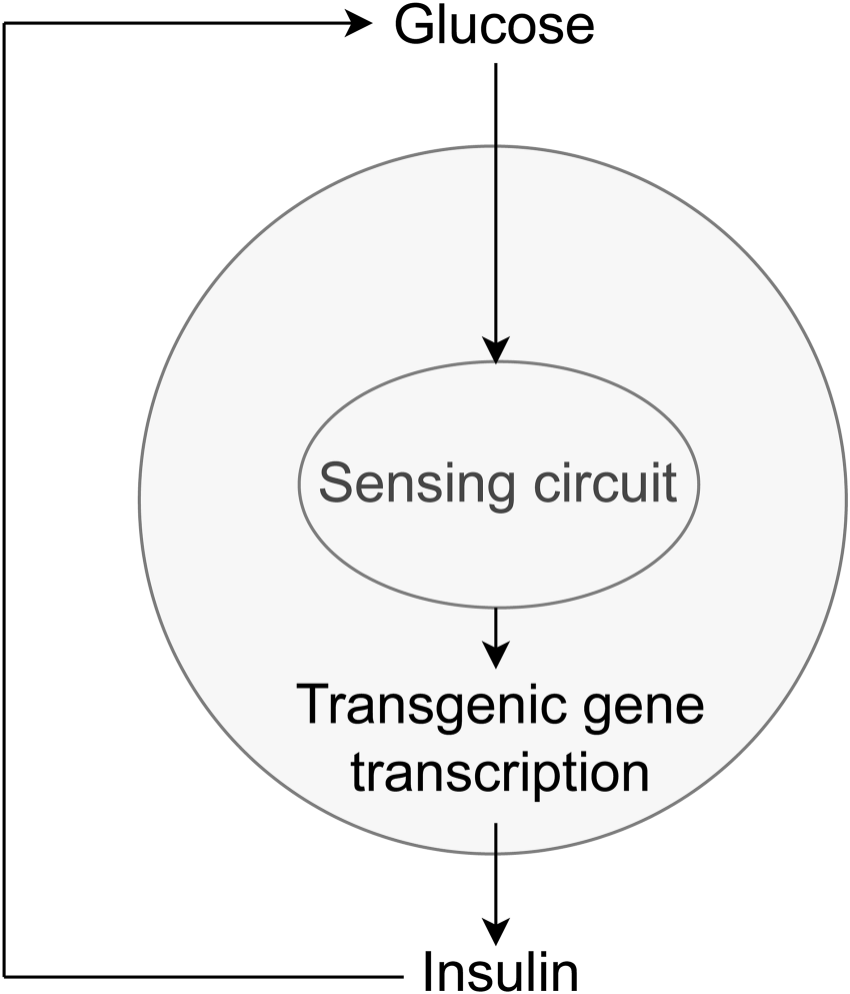
HEK-293 transgenic artificial *β*-cell, from Xie et al. [6]. Glucose levels are sensed by ion channels on the cell membrane (sensing circuits), which trigger the transcription of the transgenic insulin-producing gene and the subsequent secretion of insulin, lowering glucose blood levels.

**Fig 2:**
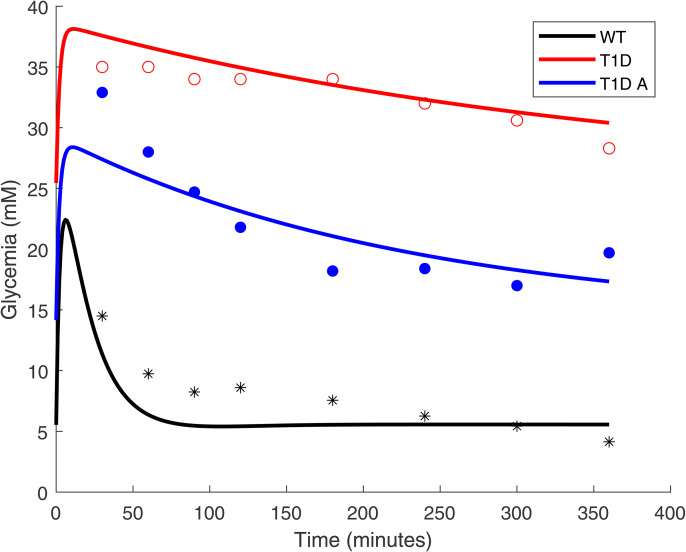
Reported results from oral glucose tolerance tests. The dots represent the mean blood glucose levels of different mice groups in a six-hour time course of an oral glucose test (as read from Xie et al. [6], Fig 4(G)). Before the oral glucose test, the mice had been fasting for an extended period of time. In the experiment, artificial *β*-cells are placed inside capsules. By diffusion, the capsules exchange insulin and glucose with the blood of the T1D mice. Red hollow dots represent the mean recorded experimental values for T1D mice without any treatment; black stars represent healthy mice; and blue solid dots represent T1D mice treated with artificial *β* cells. The solid lines are our simulation results as the reconstruction of the model presented in Xie et al. [6], Fig 4(G). Here “WT” is the healthy mice group, represented by the black curve, “T1D” is the T1D mice group without any treatment, represented by the red curve, and “T1D A” is the T1D mice group treated with artificial *β*-cells, represented by the blue curve. The cell density is set to $1.17\times 10^{7}$ cells/L to be visually consistent with experiment results; other parameters are as reported. It is evident that the artificial *β*-cell treatment has a beneficial effect on the T1D mice, however the rate of glycemia decay is not as fast as in healthy mice.

Through biochemical reactions, ion channels on the membrane of artificial *β*-cells function as a sensing circuit that controls the expression of insulin-secreting genes. The cells can roughly be viewed as a feedback controller as shown in Fig 1: when there is a high concentration of glucose in the cell’s exterior environment, the ATP concentration in the cell also increases and subsequently ion channels are triggered. The final result of the ion channel activity is the transcription of mRNA and the subsequent production and secretion of insulin to lower the blood glucose levels. After glucose levels drop, ATP levels also decrease and ion channels shut off insulin production. In Xie et al. [6], three groups of mice were used to test the effectiveness of artificial *β*-cells, healthy wild type (WT) mice, mice with T1D without any treatment, and T1D mice with artificial *β*-cell implants. In oral glucose tolerance tests using sugared water, glucose levels were measured every 30 minutes up to 150 minutes and every 60 minutes afterwards (Fig 2). T1D mice implanted with artificial *β*-cells (blue dots) showed lower blood glucose levels compared with T1D mice (red dots), but not as low as healthy mice (black dots). It appears that the glucose level in T1D mice treated with artificial *β*-cells is decreasing too slowly, failing to reach the healthy glucose range (less than 5.5 mM) [20] within two hours.

**Fig 3:**
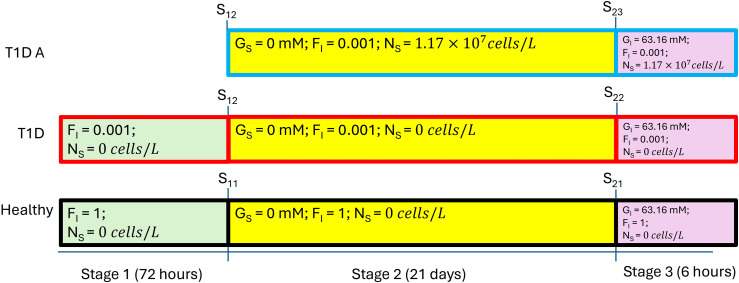
Protocol used for the oral glucose tolerance test. Initial values used in Stage 1 can be found in Table S1 and Table S5 within S1 File.

In addition to these *in vivo* experiments, Xie et al. [6] presented a unified mathematical model of the artificial *β*-cells (with details of the processes of glycolysis, ion channel transduction, transcription, translation, and secretion), the implant delivery capsules, and the mouse glucose metabolism for the *in vitro* experiments. They use that model to analyze and explain the oral glucose tolerance test results for each group of mice. We reproduced this model in Matlab, handled some vagueness, and corrected some possible errors (see S1 File). There are dozens of ordinary differential equations and variables in the model to describe the dynamics of blood glucose level and the artificial *β*-cells. Specifically, the model simulates the process of increasing ATP level in the artificial *β*-cell as a result of a high extracellular glucose level, as well as the subsequent mRNA transcription and insulin production as a result of ion channel activation for T1D mice implanted with artificial *β*-cells. The model is also capable of simulating long-lasting high glucose levels in T1D mice without any treatment as well as a changing glucose level pattern in healthy mice. Of main interest to us is the oral glucose intake test, corresponding to the experimental results which we reproduce in Fig 2. This is a three-stage protocol (cf. Ref. [6], Table S3 in S1 File, SI p. 40) as follows: Stage 1: set all variables to initial values with respect to Table S1 and Table S5 within S1 File, set the insulin production factor $F_{I}$ to 0.001 for T1D mice and 1 for healthy mice, and simulate for 72 hours without the cells implant (by setting $\gamma_{I}$ and $\gamma_{G}$ to zero). At the end of Stage 1, we reach state $S_{11}$ for healthy mice and state $S_{12}$ for T1D mice. Simulating Stage 2 involves first setting $G_{S}$ to 0 for $S_{11}$ and $S_{12}$ then continuing according to one of three scenarios: (1) With $S_{11}$ as initial condition, we simulate for 21 days for healthy mice without implant, and we reach state $S_{21}$. (2) With $S_{12}$ as initial conditions, we simulate for 21 days for T1D mice without implant, and we reach state $S_{22}$. (3) With $S_{12}$ as initial condition, we add the artificial *β*-cell implant to T1D mice, then simulate for 21 days to reach state $S_{23}$. Here, the implant is added by setting $\gamma_{I}$ and $\gamma_{G}$ to positive values according to Table S8 within S1 File and setting cell density to $1.17\times 10^{7}$ cells/L. The protocol can be represented as in Fig 3. This last simulation serves the purpose of making the artificial *β*-cells “mature”: the inner states of the cell are balanced with the outer steady-state environment. Lastly, we have Stage 3, the actual oral glucose test. We manually set $G_{I}$, the glucose level in the mouse stomach, to 63.16 mM in states $S_{21}$, $S_{22}$, and $S_{23}$, respectively, then perform three simulations. (Healthy) With $S_{21}$ as initial condition, we simulate healthy mice without any implant for six hours; the result is the black curve in Fig 2. (T1D) With $S_{22}$ as initial condition, we simulate T1D mice without any implant for six hours; the result is the red curve in Fig 2. (T1D A) With $S_{23}$ as initial condition, we simulate T1D mice with implant for six hours (cell density $1.17\times 10^{7}$ cells/L); the result is the blue curve in Fig 2. Focusing on Stage 3, after a certain amount of time, artificial *β*-cells implanted in T1D mice indeed successfully decrease glucose levels compared with T1D mice without treatment. But in general, the glucose curve of the T1D group does not follow the same pattern as the healthy group: it drops too slowly and does not reach normal range within six hours.

## Methods and results

**Fig 4:**
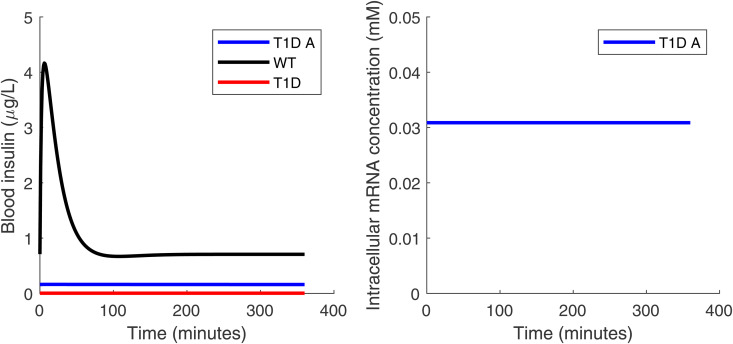
Insulin and mRNA time courses. The insulin concentration in blood (left panel) and the mRNA concentration in cells (right panel) during Stage 3 (the six-hour time course after the oral glucose test). The implanted *β*-cell density is $1.17\times 10^{7}$ cells/L. The legend is the same as for Fig 2. Notice that in the right panel there is only one curve since the intracellular mRNA concentration is only meaningful in the T1D mice group with implanted cells. The artificial *β* cells are working at their full capacity as evidenced by the high level of mRNA.

From Fig 2, it can be seen that the glucose level in T1D mice implanted with artificial *β*-cells (with density $1.17\times 10^{7}$ cells/L) is always too high after oral glucose intake, compared with healthy mice. There can be two possible causes: either there is an insufficient quantity of artificial *β*-cells implanted, or the quantity is sufficient but the cells are not functioning at their peak level for producing insulin. To understand what is the most likely cause, we plotted the mRNA concentration in the cell over the 6-hour time course of Stage 3 in Fig 4. It shows that the mRNA concentration in the cells reaches 0.03087 mM quickly and remains at that value, which is about 20 times higher than typical mRNA concentration in mammalian cells. There can be up to $10^{6}$ mRNA molecules in a single cell [21]. For the cells used in this paper with radius of 6.5 *μ*m, the cell volume is about $1.15\times 10^{-12}L$ using sphere cell shape. That gives the in-cell mRNA density as $\frac{1000\times 10^{6}}{N_{A}\times 1.15\times 10^{-12}L}≃0.0014mM$, where $N_{A}$ is the Avogadro’s constant. We interpret the simulated 0.03087 mM as indicating near-maximal transcriptional activation in the model, i.e., the synthetic cells are operating near peak capacity under these conditions.

**Fig 5:**
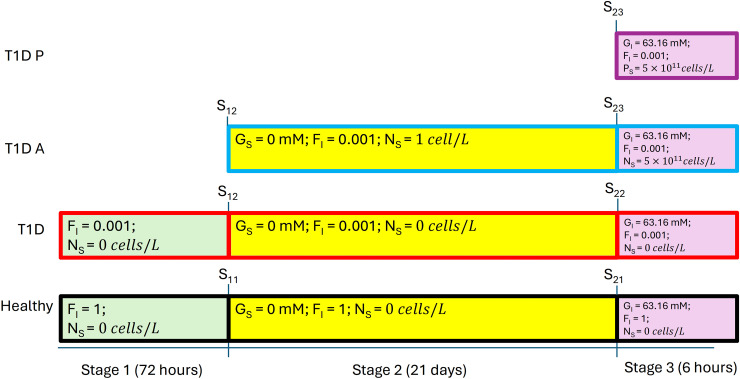
Protocol used with adjusted cell density. Here the simulated cell density has been increased to $5\times 10^{11}$  cells/L. Note that $P_{S}$ in Stage 3 means the PID *β*-cell density.

We now consider insufficient quantity of cells as a possible cause of imperfect control. To increase the quantity of cells, we can either increase the cell density or the volume. As the volume is fixed by the implantation procedure, here we vary the cell density alone. We increased the density to $5\times 10^{11}$  cells/L. We believe this is a proper number to use since it is consistent with the radius of HEK-293 cells from Supplementary material of Xie’s paper (6.5 *μ*m). Assuming spherical cells with $V_{cell}=\frac{4}{3}\pi r^{3}≃\frac{4}{3}\pi (6.5\;\mu m{)}^{3}≃1.15\times 10^{3}\;\mu m^{3}=1.15\times 10^{-12}\;L$, that gives an ideal cell density of $1/(1.15\times 10^{-12})≃8.7\times 10^{11}$ cells/L. We use a lower cell density to account for some extracellular space. This change, however, causes a problem during the second stage of the protocol: the glucose drops to an extremely low level (0.238 mM) within 20 minutes of the second stage. This is a dangerously low glucose level for mice [22]. Therefore, if we implant a high density value of the *β*-cells that is close to the healthy mice in the second stage, the mice are unlikely to survive during this stage. Hence, we changed the protocol to the following: Stage 1: same as stage 1 in the description of the artificial *β*-cell protocol above; Stage 2: the same setup for the mice without implant, but we also implant a very low density (1 cell/L) to the T1D mice such that these cells with extremely low density do not have any practical effect on the mice. After three weeks of fasting, the internal states of the artificial *β*-cells are balanced with the outer environment of the T1D mice; Stage 3: everything is the same as stage 3 in the artificial *β*-cell background session except we instantaneously increase the implanted cell density value to $5\times 10^{11}$  cells/L. This is to simulate an oral glucose test right after the implant of high density mature *β*-cells as the treatment for T1D mice. The protocol for increased cell density value can be represented as in Fig 5.

**Fig 6:**
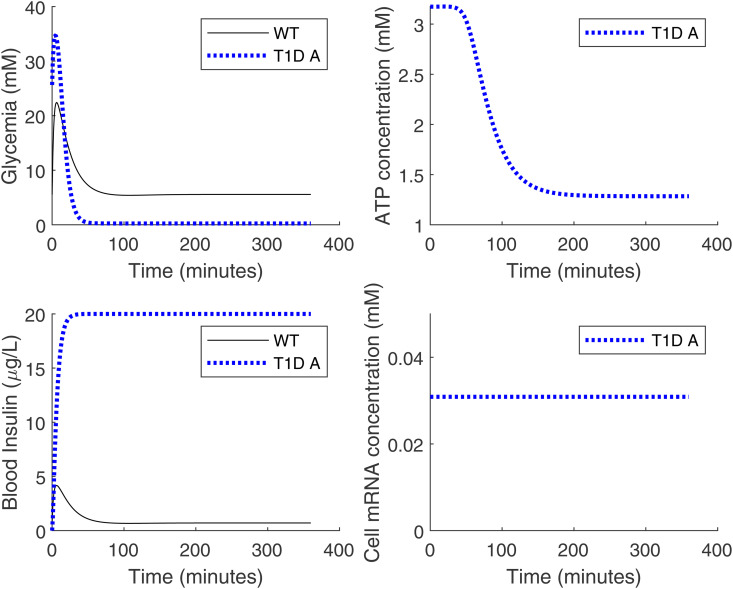
Time courses of the simulated oral glucose tolerance test with adjusted cell density. Setting the implanted artificial *β*-cell density to $5\times 10^{11}$cells/L, the upper bound for plasma insulin is set to 20*μ*g/L, which is roughly an order of magnitude higher than typical physiological levels. For comparison, mouse plasma insulin can reach about 60*μ*U/mL (2.08*μ*g/L) [23]. The legend is the same as in Fig 2. In response to the rapid drop of glucose levels when the cell density increases (upper left panel), the ATP also drops to a very low level (upper right panel). However, the artificial *β*-cells are unable to stop the production of insulin on time (lower left panel). The mRNA remains at a high level even when the ATP level is low due to the insensitivity of ion channels (lower right panel).

A plot of the results of the above-stated Stage 3 is shown in Fig 6. After increasing the cell density to $5\times 10^{11}$ cells/L, the blood insulin level can roughly reach 4 *μ*g/L within five minutes, which is comparable to that of healthy mice. However, as a result of this increase in cell density, by 50 minutes the blood glucose level drops to near zero, which again is lethal. According to the simulation, we observe that the cells are still producing insulin almost to full capacity even after the glucose concentration has reached a healthy level from Fig 6. Therefore, it is necessary to introduce some mechanism that shuts off insulin production when necessary, and this is precisely what a controller can do.

Our aim here is to explore PID control in the artificial *β*-cell. The following differential equation describes the PID controller as a conceptual replacement for the ATP-to-mRNA pathway in the PID *β*-cell:


$$\frac{dM_{S}}{dt}=(P_{g}\times (A(t)-A_{0})+I_{g}\times \int_{0}^{t}(A(z)-A_{0})\;dz+D_{g}\times \frac{dA(t)}{dt}-k_{dm}M_{S})\times \left(1-\frac{M_{S}}{0.031}\right)$$

Here, A(t) is the concentration of ATP in the cells; $P_{g}$, $I_{g}$, and $D_{g}$ represent the proportional, integral, and derivative gain respectively; $A_{0}$ is the target value for ATP and is set to 3.2 mM. (Rounding 3.1737 mM, the ATP concentration obtained in the simulation of Stage 2, after a long fasting period for T1D mice implanted with artificial *β*-cells with negligible density (one cell/L) as shown in Fig 5). The term $(1-\frac{M_{S}}{0.031})$ imposes an upper bound on the PID *β*-cell‘s mRNA concentration, ensuring it does not exceed that of the artificial *β*-cell without control from the simulation as shown in Fig 4. This ensures a fair comparison between the two.

**Fig 7:**
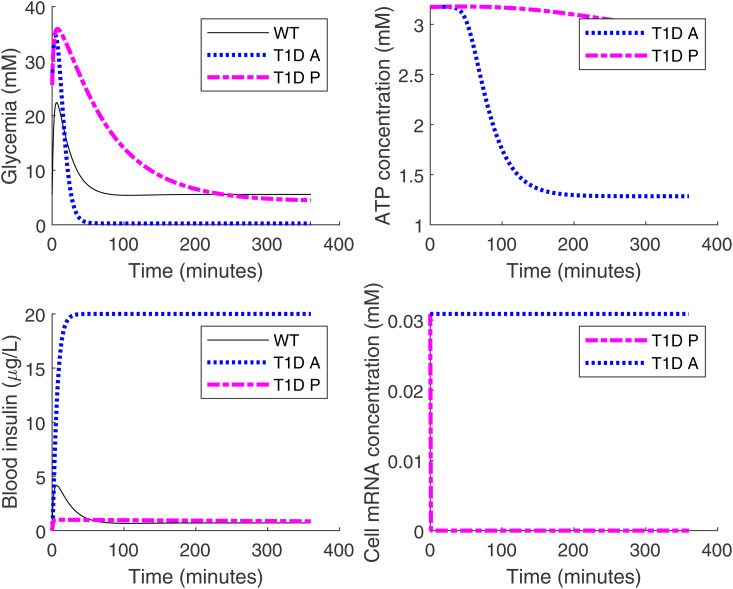
Comparison of artificial *β*-cells and PID *β*-cells. Here “T1D P” means the T1D mice treated with PID *β*-cells. The magenta dashed line represents the simulation for treatment result of T1D mice with PID *β*-cells implanted. The implanted PID *β*-cell density is the same as that of artificial *β*-cell, which is $5\times 10^{11}$ cells/L. For the PID controls, we have selected the gain parameters as $P_{g}=14$, $I_{g}=11$, and $D_{g}=6$. Other legends are the same as in Fig 2. For T1D mice treated with PID *β*-cells, the glucose level converges to a normal level (upper left panel), as does the ATP level (upper right panel). For mice treated with PID *β*-cells, the blood insulin level does not explode (lower left panel) since mRNA, the switch for insulin production, is shut off on time (lower right panel).

We must also optimize the gain parameters. We use a constrained sampling approach fixing a lower bound of 4.14 mM on the blood glucose level, which the mean blood glucose level in healthy mice after six hours of fasting, from *in vivo* experiments. We explore $P_{g}$, $I_{g}$, and $D_{g}$ from 0 to 15 with a step size of 1. We limited the gain parameters to no more than 15 since in an eventual implementation they are determined by reaction rates from the CRN and we wish to avoid large ranges of required rates. Subject to the constraint, we select the glucose curve with the minimum $L^{2}$ distance from the glucose curve of healthy mice. This is achieved with $P_{g}=14$, $I_{g}=11$, and $D_{g}=6$; the results are shown in Fig 7.

After 110 minutes, the blood glucose level of T1D mice implanted with PID *β*-cells drops below 10 mM and then remains around 5 mM throughout the experiment. The mRNA concentration in PID *β*-cells drops to zero after the first minute, and the secretion of insulin stops, as desired. Thus both hyperglycemia and hypoglycemia are avoided.

**Fig 8:**
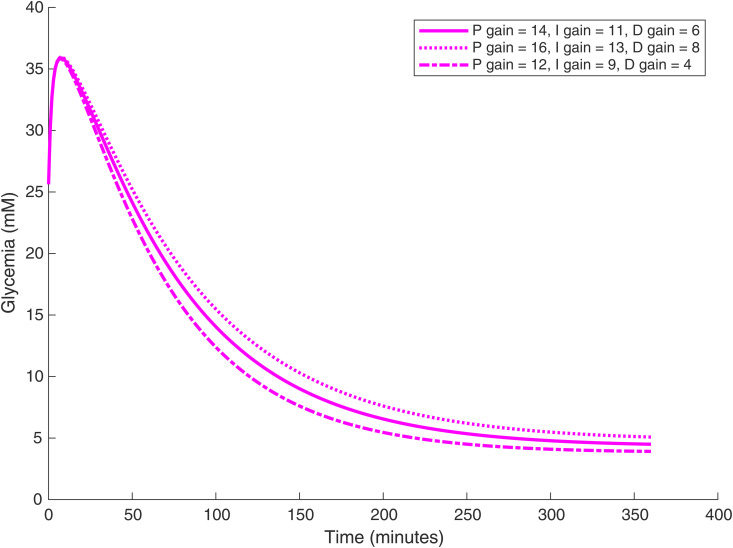
Glucose time courses for three groups of PID gain parameters. The implanted PID *β*-cell density is $5\times 10^{11}$ cells/L. It is noteworthy that when the PID gain parameters are increasing, the curve is actually converging more slowly, contrary to typical PID tuning patterns.

The PID *β*-cell also provides a more flexible tuning space compared with the artificial *β*-cell: the glucose time courses can be tuned by setting different PID gain parameters (Fig 8).

**Fig 9:**
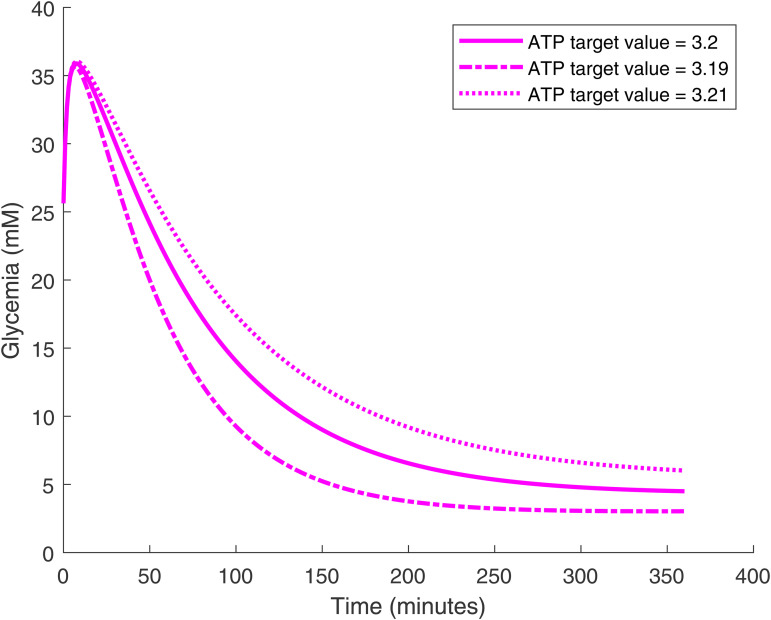
Glucose time courses as the ATP target value is varied. The implanted PID *β*-cell density is $5\times 10^{11}$ cells/L. Glucose level converges more rapidly for lower target value and vice versa.

**Fig 10:**
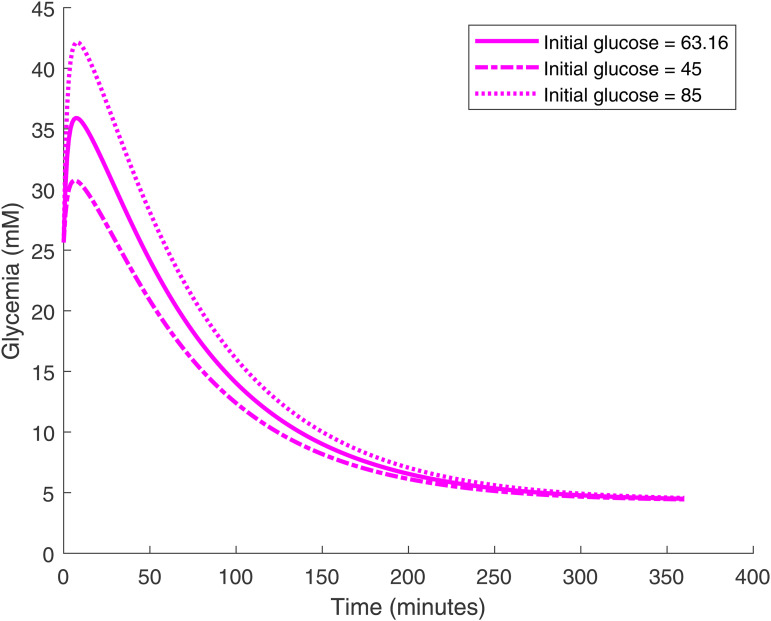
Glucose time courses as the initial stomach glucose level is varied. The implanted PID-regulated *β*-cell density is set to $5\times 10^{11}$ cells/L. In all cases, the blood glucose level consistently converges to a steady state, regardless of the initial stomach glucose level, i.e., meal size.

The long-term glucose level can be traded off against the glucose decrease rate. If we can tolerate a lower long-term glucose level (with the risk of hypoglycemia) we can set lower PID gain parameters for a faster decrease rate. However, for artificial *β*-cells, the glucose curve time course cannot be changed once the cell density is fixed. We also find that the glucose time course is highly sensitive to the target value of ATP, as shown in Fig 9. In general, with a smaller ATP target value glucose decreases faster and converges to a lower level. Furthermore, regardless of the initial stomach glucose level—which represents the meal size ingested by the mouse—the artificial *β*-cell consistently restores the blood glucose to the designated target level, as shown in Fig 10.

### Ethics statement

This study is entirely computational/simulation-based and involved no human participants, human data or tissue, and no animal subjects or experiments. Accordingly, Institutional Review Board (IRB) approval, informed consent, and Institutional Animal Care and Use Committee (IACUC) approval were not required.

## Discussion

By applying a PID control to an artificial *β*-cell, the glucose level is controlled with respect to the ATP level. By changing the gain parameters and the ATP target value, a PID controller is capable of providing a wide range of time course patterns and thus different glucose management outcomes. However, the glycemia in oral glucose test of PID *β*-cell does not reach healthy levels as quickly as in healthy mice. One possible reason is that we are controlling the glucose level indirectly by regulating the ATP level with mRNA as the actuator. Even if we can control the glucose directly, it would be difficult for the PID *β*-cell to work as well as the beta cell in healthy mice, since the beta cell in healthy mice is controlled by both the blood glucose level and the insulin level [24]. In addition, mRNA is a relatively weak actuator to control glucose levels, since mRNA does not directly affect glucose levels. In spite of the dramatic decrease in mRNA levels, insulin levels do not decrease as quickly as in healthy mice. Another limitation of the current method is the lack of glucagon regulation. In healthy mice, the glucose level is regulated by both insulin and glucagon [25], whereas our current PID controlled model considers only insulin since this is adapted from Xie’s work [6]. This simplification restricts the biological realism and limits direct clinical applicability. However, we view this work as a first-step computational proof of concept, demonstrating that an embedded PID-like control can enhance synthetic *β*-cell performance. Extending this control framework to dual-hormone control is an important direction for future work. Such an extension would better capture the counter-regulatory dynamics of glucose homeostasis and could increase the clinical relevance of synthetic *β*-cell models.

While this work remains computational, several synthetic-biology approaches suggest feasible routes to *in vivo* realization. Recent progress in chemical reaction networks and DNA strand displacement demonstrates that proportional, integral, and derivative operations can be encoded biologically [26–29]. Engineered mammalian gene circuits with tunable promoters and feedback loops may provide a route to embedding a controller in living cells. The present work may serve as an inspiration for such implementations. Although preliminary, these directions highlight possible paths toward clinically meaningful treatment approaches. In practice, combining a CRN-derived integral module, fast feed-forward/feedback motifs for approximate derivative action, and adjustable expression/degradation gains for proportional control offers a compact, modular path to PID-like regulation in mammalian cells.

PID *β*-cells can be viewed as a function with four independent variables (PID gains and ATP target values) and therefore optimization methods should be explored for parameter selection. In addition, PID control with constant gain parameters is not optimal for controlling the glucose level. The insulin production rate changes dramatically following a meal, which is a challenge for PID controllers. We plan future studies of other controller designs in search of more optimal solutions, such as variable gain parameters for PID control, fuzzy control, reinforcement learning control, and model predictive control; additionally, we will study specific implementations of control as in-cell chemical reaction networks.

To select the cell density parameter, we first calibrated it to reproduce the results of Xie et al. [6]. We found this density is insufficient to produce adequate insulin. We therefore increased the density to a reasonable, higher level (details in Methods and Results, paragraph 2) and show that at this density active control is required to prevent excessive insulin production.

In clinical practice, when implanting cells into the human body, patient-specific factors—such as sex, body mass index, and comorbidities—should also be considered. These considerations may necessitate more advanced control strategies than simple PID control presented here, so we reserve them for future work.

This study also fits into the field of diabetes management strategies. The safety of closed-loop control systems has been improved by continuous and intermittent glucose monitoring technologies [30]. The risks of daytime hypoglycemia in the closed-loop designs are also present [31]. These works highlight the clinical relevance of feedback control algorithms, to which the proposed PID framework contributes from a synthetic biology perspective. Furthermore, recent work demonstrates the value of combination pharmacological therapy (GLP-1 receptor agonists with SGLT2 inhibitor) in the control of the glucose level and body weight for T2D patients [32]. Artificial *β*-cells with dual hormone synthetic circuits embedded can also be applied in clinical settings by analogy. Hence, we do not view our work as a replacement for medical devices or other diabetic treatment, but as a complementary path that may eventually cooperate with them.

## Supporting information

S1 File Comprehensive supporting information file. Two appendices are included in the supporting information file: an Appendix on PID control background (Fig S1); and an Appendix describing the PID *β*-cell model parameter decisions (Fig S2) and fully specifying the adopted model, with a model diagram (Fig S3), ODEs, and tables of all the parameters (Tables S1–S8).
(PDF)
