# Supplementary material for: PID-controller enhanced artificial β-cells
Source: PLoS One. 2026 Mar 18;21(3):e0342799. doi: 10.1371/journal.pone.0342799 (PMC12998882; doi:10.1371/journal.pone.0342799)
Supplement: S1 File — Two appendices are included in the supporting information file: an Appendix on PID control background (Fig S1); and an Appendix describing the PID β-cell model parameter decisions (Fig S2) and fully specifying the adopted model, with a model diagram (Fig S3), ODEs, and tables of all the parameters (Tables S1–S8). (PDF) [file pone.0342799.s001.pdf]

Supporting Information for PID-controller enhanced artificial β-cells

Lin Liu, Bruna Jacobson, Darko Stefanovic

Background on PID control

In control theory, the proportional-integral-derivative controller (PID controller) is widely used to solve the problem of maintaining a desired output level when the states are subject to frequent changes in a dynamic system. In our case, the artificial β-cell is a dynamic system with ion channel as the controller whose input is the ATP and whose output is mRNA. Due to the poor performance of ion channel according to the model (main paper Fig 6), we replace it with an abstract proportional-integral-derivative controller (PID controller) that can be implemented as a chemical reaction network (CRN).

As shown in Fig S1, PID controllers are feedback control loop mechanisms widely used in applications requiring continuous modulated control. PID controllers consist of three major components: proportional operator, integral operator, and derivative operator. For an input signal X the proportional operator generates an output signal $P_g X$, where the non-negative constant value P_g is called proportional gain. The integral operator generates an output signal $I_g \int_0^t X(z)\, dz$, where the non-negative constant value I_g is called integral gain. The derivative operator generates an output signal $D_g \frac{dx}{dt}$, where the non-negative constant value D_g is called derivative gain.

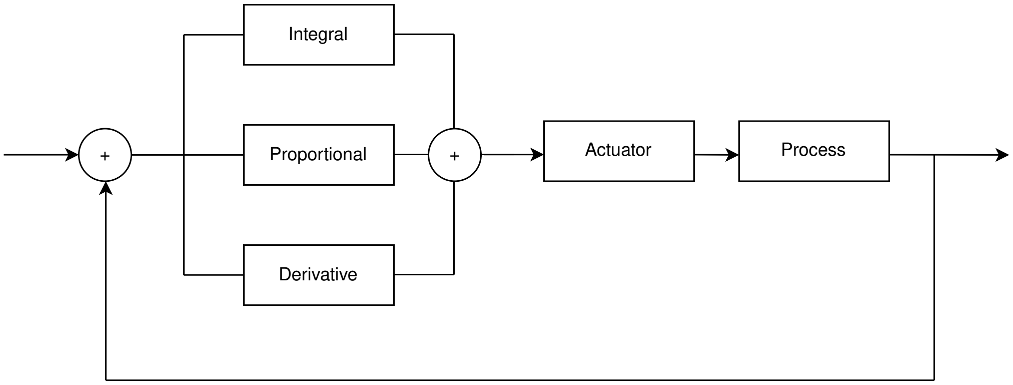

Fig S1. The PID-controller diagram

The actuator and the process (also known as plant) are two important concepts of a PID controller. Actuators, such as motors or heaters, generate force or energy to change the system state. Actuators may be linear or non-linear, but in either case when input to the actuator is zero, the actuator normally exerts little or no influence on the process. An insulin-producing gene serves as an actuator, where the input is the difference between intracellular ATP concentration and a predefined target level of ATP. When this difference value is zero or negative, the expression of the

insulin-producing genes is suppressed such that the secreted insulin amount is also minor. The process is the value that the actuator is trying to affect and control, the blood glucose concentration in our case. The process can be modeled as a time function X. In PID controllers, we have a benchmark value $X_{\mathbf{benchmark}}$ (also known as the set point) for the process. This is the desired target value we want our process (blood glucose level) to be. The error term, which is defined as $X - X_{\mathbf{benchmark}}$, is consistently calculated and used as the input signal to the proportional operator, integral operator, and differential operator. The output of these three components is summed and fed into the actuator to control the process. Over time, the error term changes as the process changes. The new error term as a time function is fed into the proportional operator, integral operator, and differential operator again in a feedback loop. When X approaches $X_{\mathbf{benchmark}}$, the output from the proportional operator approaches zero. Hence, the target value cannot be achieved by feeding only the output from the proportional operator into the actuator. This is called the steady state error. Integral operators can remedy the steady state error by effectively accumulating the error term and adding it to the proportional output. In this way, the actuator can maintain a higher output level even if X is approaching $X_{\mathbf{benchmark}}$. But this brings another issue: the error term is still a relatively large value even if $X = X_{\mathbf{benchmark}}$. This causes an overshoot of the process: X goes beyond $X_{\mathbf{benchmark}}$, either lower or higher, and fluctuates around $X_{\mathbf{benchmark}}$ for a relatively long time before it converges to $X_{\mathbf{benchmark}}$. The derivative operator can minimize this overshoot and let X converge faster to $X_{\mathbf{benchmark}}$ [1].

The Ziegler-Nichols tuning method is often used to experimentally find the PID controller parameters. In this method, the integral and derivative gains are first set to zero. Then we slowly increase the proportional gain until the process we are trying to control begins to oscillate. Then we gradually increase the integral and derivative gains to deal with the overshoot. However, this method fails in our setting. We tried to gradually increase the proportional gain but failed to observe any oscillation. And the process (the glucose level in our case) normally converges to the target value faster when the PID parameters are larger, which also is not observed in our case (main paper Fig 8).

In the face of failure of standard methods we use the constrained sampling approach described in the methods and results section.

On reproducing the model of artificial β-cells

We encountered the following issues when trying to reproduce the results of Xie et al. [2].

There is a term called virtual compartment in this paper. In light of the lack of details provided in the paper, it is unclear what the purpose of the virtual compartment is.

The model also has multiple tunable parameters: the initial value of glucose level, the artificial β-cell density, the vascular exchange constants (positive means the implanting of the cell, zero means there is no implanting), diabetic factor (zero means fully T1D mice, one means fully healthy mice). This model also involves many biology-related constants. We reconstructed this model in MATLAB and followed

exactly the same protocols described in Ref. 2, however we encountered the following uncertainties and errors on some of these parameters and constants.

In equation (17) of the supplementary material of Ref. 2, a metabolic rate term $R_4(Ca_{Si})$ is defined as $\frac{V_{max4}(Ca_{Si})^{d_4}}{K_{m4}^{d_4}+(Ca_{Si})^{d_4}}$. However, we fail to find the d_4 value in the main paper or the supplementary material of Ref. 2. In equation (6) and equation (7) of the supplementary material of Ref. 2, a mathematical model for the synthetic compartment is described. Both equations contain a term $I_{NaK}(Na_{Si})$; however, the definition of this term is missing from Ref. 2. Equation (34) of supplementary material of Ref. 2 describes the blood insulin concentration rate of change. In this equation, there is a term $\xi(I)$, which is defined as $\frac{I^{n_{HI}}}{K_{MI}^{n_{HI}}+I^{n_{HI}}}$ on top of page 14 of Ref. 2. The values of K_{MI} and n_{HI} are missing. Also in this equation(34), there is an F_I factor indicating the mice insulin production. This factor is either 0 or 1, according to Table S10 of the supplementary material of Ref. 2, where 0 means a fully diabetic mouse and 1 indicates a fully healthy mouse. But in the legend to Figure S13(D) of the supplementary material of Ref. 2, there is a variable of "native insulin production" ranging from 0.1% to 100%. We are not sure if the "native insulin production" corresponds to the F_I factor. And in Equation (16) of the supplementary material of Ref. 2, the unit on the right hand side does not match the left hand side. Also there is a variable N_S indicating the cell density value defined in Table S4 of the supplementary material of Ref. 2. This variable takes multiple values throughout the paper and the default value is 7.84×10^8 cells/L as reported in Table S4 of the supplementary material of Ref. 2. In Figure 4G of the main paper of Ref. 2, there is a description of a total of 5×10^6 cells implanted into the mice, but no data on the cell density is provided. We find the volume of the total capsules where the artificial β-cells located is 3.35×10^{-4} L, indicating the cell density should be 1.49×10^{10} cells/L. However, neither of these two density values can match what is reported in Figure 4G of the main paper of Ref. 2 via simulation. Both density values are too high (Fig S2). We contacted the authors of Ref. 2 regarding these issues, but have not received concrete answers. Hence here we have tried to correct these equations and make guesses with respect to the missing parameters consistent with the reported experimental results.

The following are our corrections and guesses. For the virtual compartment, we believe it to simulate the stomach, since G_I is described with this equation: $\frac{dG_I}{dt} = 0.2986 \times (G - G_I)$, where G_I is the glucose concentration in the virtual compartment and G is the blood glucose concentration. The equation indicates in the virtual compartment, glucose is diffused into the bloodstream, just as the stomach does following a meal. There is a d term in Table S9 of the supplementary material of Ref. 2 with a value of 1.6. The term is described as "Hill coefficient for calcium-dependent gene expression", which is identical to equation (17) of the supplementary material of Ref. 2. This term is not used anywhere in the paper. Our guess is the d_4 parameter in equation (17) from the supplementary material of Ref. 2 is a typo and it should be d instead. For the $I_{NaK}(Na_{Si})$ term, we guess this is also a typo and use the clearly defined $I_{NaK}(V, Na_{Si})$ term instead. We find a same $\xi(I)$ term from the supplementary material (page 27) of a reference [3] cited in Xie's paper, where $K_{MI} = 10^{-2} ng/mL$ and $n_{HI} = 8$. We adopted these two values in the model.

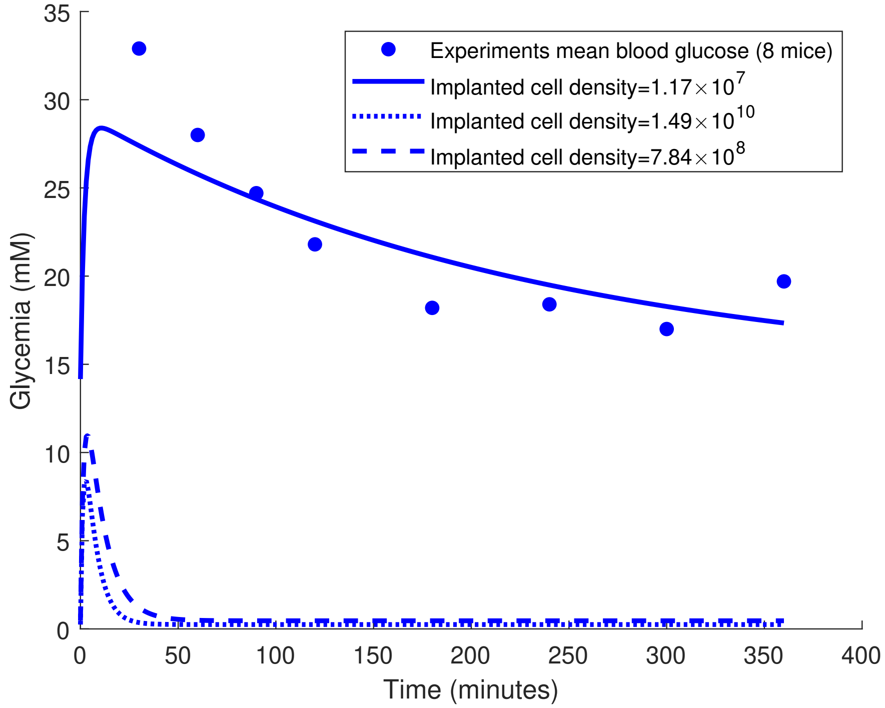

Fig S2. Glucose curve for different cell density values. Various values are tried and 1.17×10^7 cells/L gives visually consistent results with Figure 4(G) of Xie et al. [2]. It appears that the other two density values as described in Xie et al. [2] are too high and we fail to reconstruct their Figure 4(G) with these two values.

We believe “1 or 0" is also a misleading description for term F_I. We determined the correct description should be “0 to 1" and we choose this value to be 0.1% for T1D mice. As for equation(16) in the supplementary material of Ref. 2, we changed it to $R_3(6CP, A) = V_{max3} \times \frac{K_{m3}^{2c} 6CP}{K_{m3}^{2c+1} + A^{2c+1}} \times (1 - \frac{A}{A_{tot}})$, such that the dimensions are the same on both sides of this equation. We tuned the cell density parameter such that the model's simulation results can match those reported in Figure 4G of Ref. 2 as shown in Fig 3 of the main paper.

The adopted model

Equations of the artificial β-cell mathematical model in this paper are listed. Most of them are as in the supplementary material of Xie et al., Ref. 2. Equations that were modified, as described above, are marked with **, and the assumptions we made are

Table S1. The glucose and insulin related variables

Name	Definition	Unit	Initial value
G	Concentration of glucose in the blood	mM	0
G_I ***	Concentration of glucose in an additional virtual compartment	mM	0
I_a	Concentration of insulin in an action compartment	μg/L	0
I_i	Concentration of insulin in a virtual compartment	μg/L	0
I	Concentration of insulin in the blood	μg/L	0

marked with ***. The glucose and insulin metabolism can be described as:

$$\frac{dG}{dt} = g_p - (g_r + k_{si} I_a)\, G - \frac{V_S}{V_B}\gamma_{GS} - \frac{V_B}{V_G}\gamma_{GI} \tag{1}$$

$$\frac{dG_I}{dt} = \gamma_{GI} \tag{2}$$

$$\frac{dI_a}{dt} = I - k_{iar} I_a \tag{3}$$

$$\frac{dI_i}{dt} = F_I\,(G - k_{iir} I_i) \tag{4}$$

$$\frac{dI}{dt} = F_I \left[k_p G + k_i I_i + k_d \xi(I) \frac{dG}{dt} \right] - \frac{V_S}{V_B}\gamma_{IS} - k_{ir} I \tag{5}$$

$$\xi(I) = \frac{I^{n_{HI}}}{K_{MI}^{n_{HI}} + I^{n_{HI}}} \tag{6}$$

The cell metabolic rates can be described as:

$$R_1\,(G_S) = \frac{V_{\max 1} G_S}{G_S + K_{m1}} \tag{7}$$

$$R_2\,(G_{Si}, A) = \frac{K_{m2A}^{2b-a} A^a}{K_{m2A}^{2b} + A^{2b}} \times \frac{V_{\max 2} G_{Si}}{G_{Si} + K_{m2G}} \tag{8}$$

$$R_3(6CP, A) = V_{\max 3} \times \frac{K_{m3}^{2c} 6CP}{K_{m3}^{2c+1} + A^{2c+1}} \times \left(1 - \frac{A}{A_{tot}}\right) ** \tag{9}$$

$$R_4\,(Ca_{Si}) = \frac{V_{\max 4}\,(Ca_{Si})^{d_4}}{K_{m4}^{d_4} + (Ca_{Si})^{d_4}} \tag{10}$$

$$\delta(A) = u\,\frac{A^2}{K_\delta^2 + A^2} \tag{11}$$

Table S2. The glucose and insulin related constants

Name	Definition	Value	Unit	Source
g_p	Glucose production	0.1061	mM/min	Estimated
g_r	Insulin-independent glucose removal	4.1×10^{-3}	mM/min	Estimated
k_{si}	Insulin sensitivity	1.2×10^{-3}	L/μg/min	Estimated
V_S	Volume of the synthetic compartment	1.5×10^{-4}	L	Fixed
V_B	Volume of the mouse blood	3.35×10^{-4}	L	Fixed
k_{iar}	Insulin clearance from action compartment	0.0566	1/min	Estimated
k_{iir}	Insulin clearance from virtual compartment	0.0351	1/min	Estimated
F_I ***	Insulin production factor of T1D mice	0.001	-	Fixed
F_I ***	Insulin production factor of healthy mice	1	-	Fixed
k_p	Basal insulin production rate	4.0338×10^{-5}	μg/L/mM/min	Estimated
k_i	Insulin mediated insulin production rate	1.05×10^{-10}	μg/L/mM/min	Estimated
k_d	Glucose mediated insulin production rate	0.234	μg/L/mM/min	Estimated
K_{MI} ***	Base concentration of insulin	0.01	μg/L	3
n_{HI} ***	Hill coefficient	8	-	3

Table S3. The rates related variables

Name	Definition	Unit
R_1	Rate of cellular glucose uptake mediated by Glut1	mM/min/cell
R_2	Rate of intracellular glucose and ATP consumption	mM/min
R_3	Rate of intermediate metabolites consumption	mM/min
R_4	Rate of Ca^{2+}-dependent intracellular SEAP production	mM/min
δ	Rate of global ATP consumption by the cell	mM/min

Table S4. The rates related constants

Name	Definition	Value	Unit	Source
q	Autocatalytic stoichiometry	1	-	Estimated
$V_{\max 1}$	Maximum specific rate of cellular glucose uptake	10^{-13}	mmol/min/cell	4
K_{m1}	Michaelis-Menten constant for the uptake of glucose	0.31	mM	Estimated
$V_{\max 2}$	Max rate of intracellular glucose and ATP consumption	10	mM/min	Estimated
K_{m2A}	Affinity constant for allosteric inhibition of ATP consumption by ATP	1.61	mM	Estimated
K_{m2G}	Michaelis-Menten constant for glucose consumption	1.95	mM	Estimated
a	Hill coefficient for binding of ATP to phosphofructokinase	1	-	Estimated
b	Hill coefficient for allosteric inhibition of ATP consumption by ATP	1	-	Estimated
c	Hill coefficient for allosteric inhibition of intermediate metabolites consumption by ATP	1	-	Estimated
$V_{\max 3}$	Max. intermediate consumption rate	9.96	mM/min	Estimated
K_{m3}	Affinity for intermediary consumption inhibition by ATP	1.83	mM	Estimated
A_{tot}	Total concentration of ATP + ADP	5	mM	5
u	Max global rate of ATP consumption	0.12	mM/min	Estimated
K_{δ}	Michaelis-Menten constant for ATP consumption	2	mM	Estimated
d_4 ***	Hill coefficient for calcium-dependent gene expression	1.6	-	Estimated

The ion channels in the cell can be described as:

$$\frac{dG_S}{dt} = -N_S R_1(G_S) + \gamma_{GS} \tag{12}$$

$$\frac{dG_{Si}}{dt} = \frac{1}{V_C} R_1(G_S) - R_2(G_{Si}, A) \tag{13}$$

$$\frac{d6CP}{dt} = R_2(G_{Si}, A) - R_3(6CP, A) \tag{14}$$

$$\frac{dA}{dt} = -R_2(G_{Si}, A) + 2R_3(6CP, A) - \delta(A) \tag{15}$$

$$\frac{dK_{Si}}{dt} = -\alpha \left[I_K(V, K_{Si}) + I_{KV}(V, K_{Si}) + I_{KATP}(V, K_{Si}, A) \right] + 2\alpha I_{NaK}(V, Na_{Si}) \text{***} \tag{16}$$

$$\frac{dNa_{Si}}{dt} = -\alpha I_{Na}(V, Na_{Si}) - 3\alpha I_{NaK}(V, Na_{Si}) \text{***} \tag{17}$$

$$\frac{dCa_{Si}}{dt} = -\frac{\alpha}{2} f_{Ca} \left[I_{Ca}(V, Ca_{Si}) + I_{CaV}(V, Ca_{Si}) + I_{CaP}(V, Ca_{Si}) \right] \tag{18}$$

$$\frac{dV}{dt} = -\frac{1}{C} \left[I_K(V, K_{Si}) + I_{KV}(V, K_{Si}) + I_{KATP}(V, K_{Si}, A) + I_{Na}(V, Na_{Si}) \right. \tag{19}$$

$$\left. + I_{NaK}(V, Na_{Si}) + I_{Ca}(V, Ca_{Si}) + I_{CaV}(V, Ca_{Si}) + I_{CaP}(V, Ca_{Si}) \right] \tag{20}$$

$$\frac{dM_S}{dt} = n_{NFAT} n_S R_4(Ca_{Si}) - k_{dm} M_S \tag{21}$$

$$\frac{dI_S}{dt} = F_{SCI} k_{tl} N_S M_S - k_{di} I_S + \gamma_{IS} \tag{22}$$

For a PID β-cell, equation (21) is replaced by:

$$\frac{dM_S}{dt} = (P_g \times (A - A_0) + I_g \times \int_0^t (A - A_0)\, dz + D_g \times \frac{dA}{dt} - k_{dm} M_S) \times (1 - \frac{M_S}{0.031})$$

Table S5. The ion related variables

Name	Definition	Unit	Initial value
N_S	Cell density in the synthetic compartment	cells/L	0
G_S	Concentration of glucose in the synthetic compartment	mM	0
G_{Si}	Concentration of glucose in the cell	mM	0
6CP	Intermediate metabolites (phosphorylated six-carbon sugars) concentration in the cell	mM	0
A	Concentration of ATP in the cells	mM	0.1
Ca_S	Concentration of calcium ions in the synthetic compartment	mM	1.8
Ca_{Si}	Concentration of calcium ions in the cells	mM	2×10^{-5}
K_S	Concentration of potassium ions in the synthetic compartment	mM	5.3
K_{Si}	Concentration of potassium ions in cells	mM	200
Na_S	Concentration of sodium ions in the synthetic compartment	mM	155
Na_{Si}	Concentration of sodium ions in cells	mM	8
V	Transmembrane voltage $\phi_{in} - \phi_{out}$	mV	-90
M_S	Concentration of insulin mRNA in the cells	mM	0
I_S	Concentration of insulin in the synthetic compartment	μg/L	0

The currents and potentials in the model can be described as:

$$V_K(K_{Si}) = 10^3 \frac{RT}{F} \ln\left(\frac{K_S}{K_{Si}}\right) \tag{23}$$

$$V_{Na}(Na_{Si}) = 10^3 \frac{RT}{F} \ln\left(\frac{Na_S}{Na_{Si}}\right) \tag{24}$$

$$V_{Ca}(Ca_{Si}) = 10^3 \frac{RT}{F} \ln\left(\frac{Ca_S}{Ca_{Si}}\right) \tag{25}$$

$$I_K(V, K_{Si}) = g_K(V - V_K(K_{Si})) \tag{26}$$

$$I_{KV}(V, K_{Si}) = g_{KV} \frac{1}{1 + e^{(-15-V)/5.6}} \frac{1}{1 + e^{(-43-V)/(-4.1)}} (V - V_K(K_{Si})) \tag{27}$$

$$I_{KATP}(V, K_{Si}, A) = g_{KATP} \frac{1 + (D/K_1)^{d_K}}{1 + (D/K_1)^{d_K} + (A/K_2)^{d_K}} (V - V_K(K_{Si})) \tag{28}$$

$$D = A_{tot} - A \tag{29}$$

$$I_{Na}(V, Na_{Si}) = g_{Na}(V - V_{Na}(Na_{Si})) \tag{30}$$

$$I_{Ca}(V, Ca_{Si}) = g_{Ca}(V - V_{Ca}(Ca_{Si})) \tag{31}$$

$$I_{CaV}(V, Ca_{Si}) = n_C \cdot g_{CaV}(V - V_{Ca}(Ca_{Si})) \tag{32}$$

$$I_{NaK}(V, Na_{Si}) = I_{NaK}^{\max} \frac{K_S}{k_K + K_S} \frac{Na_{Si}}{Na_{Si} + k_{Na}} \frac{V + 150}{V + 200} \tag{33}$$

$$I_{CaP}(V, Ca_{Si}) = I_{CaP}^{\max} \frac{Ca_{Si}}{Ca_{Si} + 2 \cdot 10^{-4}} \tag{34}$$

Table S6. The rates related constants

Name	Definition	Value	Unit	Source
N_S	Cell density in the synthetic compartment	*	cells/L	Estimated
V_C	Volume of HEK-293 cell	$\frac{4\pi}{3}(6.5\times10^{-5})^3$	L	Fixed
α	Coefficient converting current in pA to concentration per unit time in mM/min	$\frac{60\times10^{-9}}{V_C F}$	mM/min/pA	Fixed
f_{Ca}	Fraction of cytosolic calcium that is free	10^{-3}	-	5
C	Capacitance of HEK-293 cell	1.2×10^{-4}	nF	6
n_{NFAT}	Number of NFAT repeats for calcium dependent Insulin expression	9	-	Estimated
n_S	Plasmid copy number for Insulin transfection	3.6	-	Estimated
k_{dm}	Degradation constant mRNA	2.8×10^{-3}	1/min	Estimated
k_{di}	Degradation constant insulin in the synthetic compartment	1.1×10^{-3}	1/min	Estimated
k_{tl}	Common translation constant	5×10^{-8}	1/min	Estimated
F_{SCI}	Scaling factor insulin	0.0229	$\mu g/U$	Estimated
A_0	Target ATP value of the PID controller	3.2	mM	Fixed
P_g	Proportional gain parameter of the PID controller	*	-	Fixed
I_g	Integral gain parameter of the PID controller	*	-	Fixed
D_g	Derivative gain parameter of the PID controller	*	-	Fixed

* As described in the main paper

Table S7. The flux transfer related constants

Name	Definition	Value	Unit	Source
R	Ideal gas constant	8.3144	J/mol/K	Fixed
T	Temperature	310	K	Fixed
F	Faraday constant	9.6485×10^4	C/mol	Fixed
g_K	Conductance of potassium ions through background potassium channels	1.97×10^{-2}	nS	Estimated
g_{KV}	Conductance of potassium ions through voltage activated potassium channels	1.24	nS	Estimated
g_{KATP}	Conductance of potassium ions through ATP dependent potassium channels	1.96	nS	Estimated
g_{Na}	Conductance of sodium ions through background sodium channels	4.37×10^{-2}	nS	Estimated
g_{Ca}	Conductance of calcium ions through background calcium channels	6.43×10^{-3}	nS	Estimated
g_{CaV}	Conductance of calcium ions through voltage activated calcium channels	0.8	nS	Estimated

The cell flux transfer can be described as:

$$\gamma_{IS} = \gamma_I (I - I_S) \tag{35}$$

$$\gamma_{GS} = \gamma_G (G - G_S) \tag{36}$$

$$\gamma_{GI} = D_G (G - G_I) \tag{37}$$

Table S8. The flux transfer related constants, continued

Name	Definition	Value	Unit	Source
n_C	plasmid copy number for Cav1.3 transfection	3.6	-	Estimated
K_K	Rate constant of potassium uptake from the medium by NaK pump	1	1/min	7
I_{NaK}^{max}	Maximum sodium-potassium pump current	42.72	pA	Estimated
I_{CaP}^{max}	Maximum calcium pump current	9.4	pA	Estimated
γ_I	Vascular exchange constant insulin	0.9137	1/min	Estimated
γ_G	Vascular exchange constant glucose	0.7074	1/min	Estimated
D_G	Vascular exchange constant glucose	0.2986	1/min	Estimated
A_{tot}	Total concentration of ATP + ADP	5	mM	5

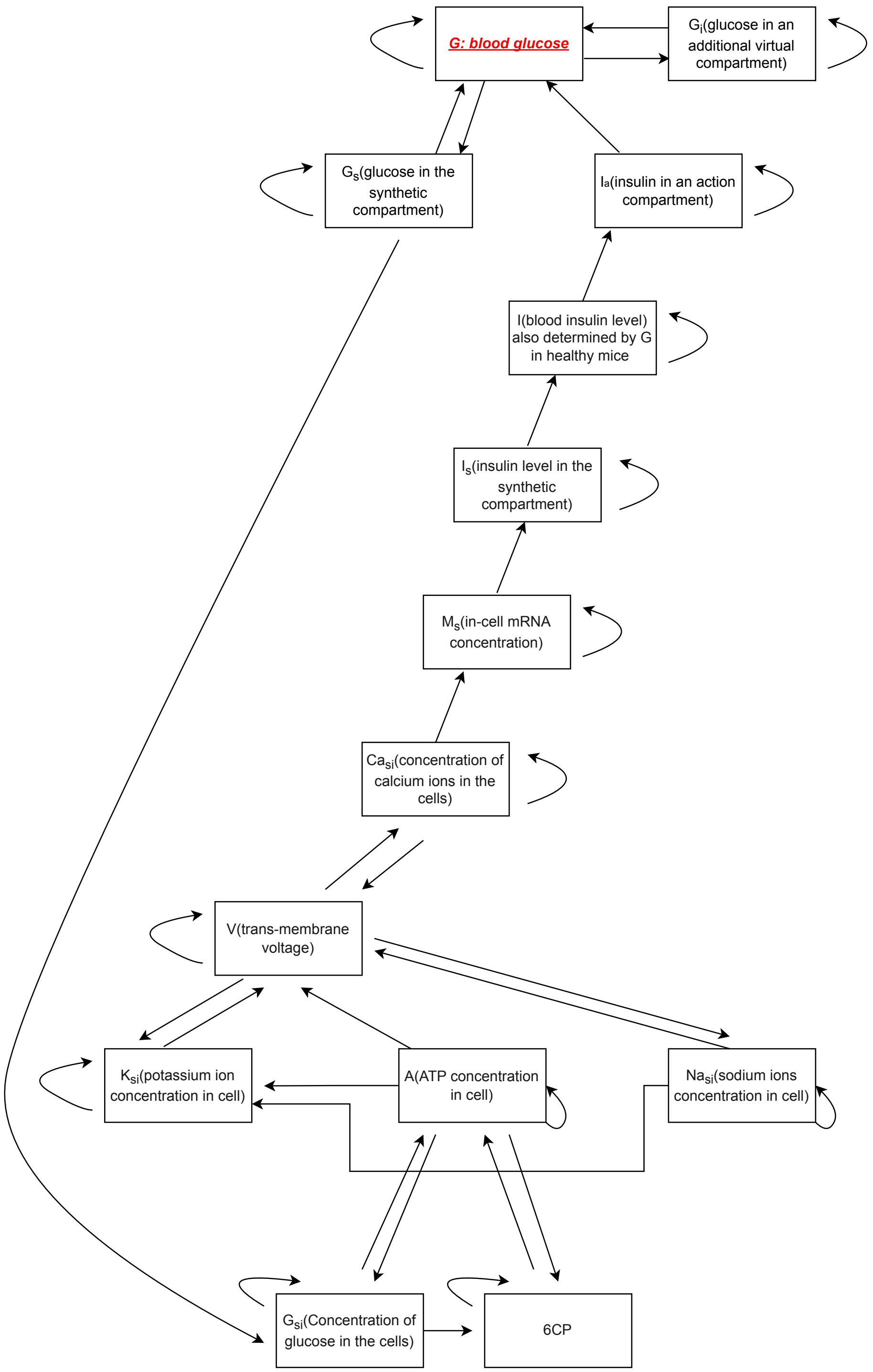

Fig S3. Directed interaction diagram for the artificial β-cell ODE model: nodes are state variables (glucose, insulin, ionic and transcriptional states), and arrows indicate which variables enter the right-hand side of another variable's ODE.

References

1. Visioli A. Practical PID control. Advances in industrial control. Springer Press; 2006.

2. Xie M, Ye H, Wang H, Charpin-El Hamri G, Lormeau C, Saxena P, et al. β-cell–mimetic designer cells provide closed-loop glycemic control. Science. 2016;354(6317):1296-301.

3. Ausländer D, Ausländer S, Charpin-El Hamri G, Sedlmayer F, Müller M, Frey O, et al. A synthetic multifunctional mammalian pH sensor and CO2 transgene-control device. Molecular cell. 2014;55(3):397-408.

4. Wertheimer E, Sasson S, Cerasi E, Ben-Neriah Y. The ubiquitous glucose transporter GLUT-1 belongs to the glucose-regulated protein family of stress-inducible proteins. Proceedings of the National Academy of Sciences. 1991;88(6):2525-9.

5. Keizer J, Magnus G. ATP-sensitive potassium channel and bursting in the pancreatic beta cell. A theoretical study. Biophysical Journal. 1989;56(2):229-42.

6. Farrell B, Do Shope C, Brownell WE. Voltage-dependent capacitance of human embryonic kidney cells. Physical Review E. 2006;73(4):041930.

7. Lindblad D, Murphey C, Clark J, Giles W. A model of the action potential and underlying membrane currents in a rabbit atrial cell. American Journal of Physiology-Heart and Circulatory Physiology. 1996;271(4):H1666-96.
